# Sacrococcygeal Teratoma: A Case Report

**DOI:** 10.31729/jnma.5230

**Published:** 2020-07-31

**Authors:** Hari Kishor Shrestha, Roshan Gongal Shrestha

**Affiliations:** 1 Department of Radiology, Om Hospital and Research Centre, Chabahil, Kathmandu, Nepal; 2 Department of Radiology, Om Hospital and Research Centre, Chabahil, Kathmandu, Nepal

**Keywords:** *coccyx*, *sacrococcygeal*, *teratoma*, *tumors*

## Abstract

Sacrococcygeal teratoma is rare and happens in 1:35,000 to 40,000 live births. It is more common in girls than boys with the reported ratio of 3:1 to 4:1. We herein report an unusual case of a huge sacrococcygeal teratoma, which was more than half of the size and weight of the baby which was terminated at 24 weeks of gestation.

## INTRODUCTION

Sacrococcygeal teratoma, which is mostly present in infancy, is a tumour located at the base of coccyx. It is thought to be derived from embryonic germ cell layers.^1,2^ The etiology is unknown. The tumors have multiple tissue types as it is composed of two or three germ cell layers. Though SCT are more prevalent in neonates, infants and children, it has been reported in adult population also. Mostly, sacrococcygeal tumours are cystic and benign whereas malignant tumours comprises 1-2%.^3,4^ Due to high vascularity in some solid tumours, it can cause problem during pregnancy and the postoperative period. Prenatal and perinatal complications may occur more commonly in fetus with sacrococcygeal teratoma. The natural history and pathophysiology of fetal SCT is different than that of postnatally diagnosed SCT. With optimal obstetric and surgical management as well as anticipation and recognition of pathophysiologic events, fetuses can be managed to survive. In a subset of fetuses with SCT, fetal resection of tumor may offer the only hope for survival.

## CASE REPORT

**Figure 1.**
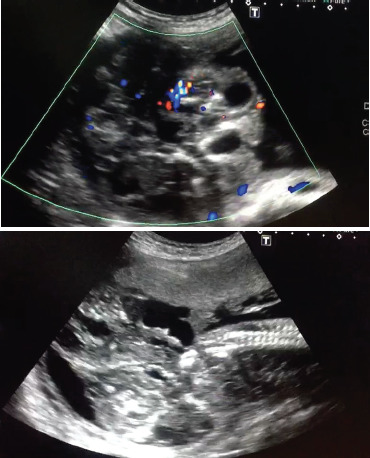
Anomaly Scan.

**Figure 2.**
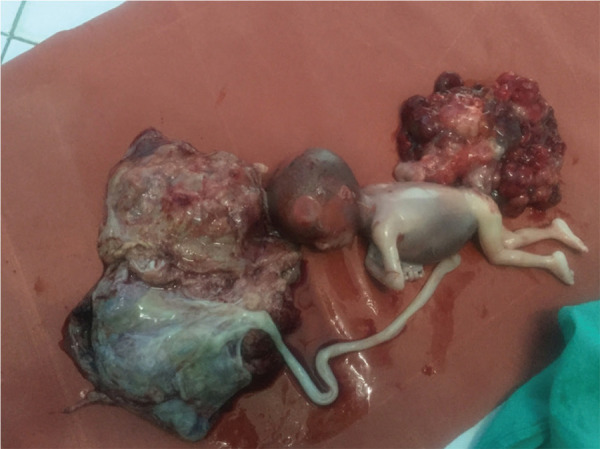
Dead Fetus with huge sacrococcygeal teratoma.

A 19 years old primigravida at 24 weeks gestation was referred for anomaly scan in our center. She had her regular ANC visit in another hospital outside Kathmandu. In anomaly scan , a large space-occupying lesion measuring 123 × 107 × 126 mm(Volume = 870cc) in right side of fetus with high vascularity, and solid component seen to be attached to posterior side of fetus near by sacrococygeal area (Figure 1). She had slightly polyhydroamnios (deepest liqour pool-8.4cm) with cystic and solid components. and other parameters (head circumference, femur length, cardiac activity) corresponding to the gestational age was within normal limits. She was then sent to Obstetrician for further management. She was hemodynamic ally stable and all biochemical investigations (hemogram, complete blood count, renal function tests, kidney function tests, blood sugar, urine examination,PT/INR and ECG) were normal. In view of large sacrococcygeal teratoma she underwent hysterotomy under spinal anesthesia. A baby girl was delivered with huge mass (Figure 2) . And polyhydraminos of around 800ml was noted. Cord blood was sent for karyotyping.

Histopathology report of the specimen confirmed immature sacrococcygeal teratoma of grade three with lymphovascular and peri-neural invasions not identified. (Table 1.)

**Table 1:** Histopathology Report.

Specimen	Placenta, sacrococcygeal tumor and fetus
Gross Description	Specimen consists of featus measuring 18×5.5 cm with attached umblical cord measuring 29×1 cm with unremarkable cut surface. Multiple fragments of placental tissue with one of them showing cord insertion is identified. Together placenta measures 19.5×16 cm. Cut surface looks unremarkable. The separate piece of tumor measures 13.5×12 cm and cut surface shows hemorrhagic and necrotic areas as well as solid gray areas with myxoid features. Sections: A-C, cord; D-E, placental tissue; F-G, sacrococcygeal area of the fetus; H-J, placenta; K-R, tumor
Microscopic Description	Sections show a tumor composed of immature neuroeptihelial elements arranged in sheets, lobules, tubules and rossettes. Tumor cells show scanty cytoplasm and oval to round hyperchromatic nuclei wiht granular chromatin. Nucleoli are inconspicuous. Areas of necrosis and autolysis are seen. Umblica cord shows two arteries and a vein. Amnion shows focal squamous metaplasia. Inter and perivillous fibrin are seen in some of the sections in focal areas. Neutrophilic infiltration is seen around and within the villi.
Diagnosis	□ Immature sacrococcygeal teratoma □ Grade 3 □ Lymphovascular and perineural invasions, not identified □ Sacrococcygeal region at the tumor base of the fetus, is free of tumor □ Maximum tumor dimension, 1 3.5 cm □ Acute villitis, placenta □ Unremarkable, umblical cord

## DISCUSSION

Tissues of Sacrococcygeal teratomas arise from all the germ layers ectoderm, mesoderm, and endoderm.^5^ They are believed to arise from the remnant of the primitive streak in the coccygeal region during the early third week of gestation from the totipotential cells of Hensen's node. The first case of sacrococcygeal teratoma has been reported on a Chaldean cuneiform tablet dated approximately 2000 BC.^6^ According to Keslar et al reports, 69 (62%) of the 96 sacrococcygeal teratomas in their series were composed of both solid and cystic elements.^7^ The cystic type may be filled with various components such as serous fluid, mucoid, or sebaceous material and are lined by true epithelium. The most common site for sacrococcygeal teratoma is coccygeal region followed by less common sites such as mediastinum, testes, retroperitoneum, brain, head and neck, vagina, stomach, and pineal region.^7^ They present mostly during the gestation period of between the 22nd and the 34th week of gestation. Sacrococcygeal teratoma when diagnosed on routine sonograms is linked with complications related to perinatal and preenatal period.^8^

Depending on the location and extension, clinical manifestations of the SCT may present with low back pain, bowel or urinary symptoms and venous engorgement of the lower limbs which may be related to the mass effects of the tumour. In malignant lesions, markers of biochemical origin such as AFP, carcinoembryonic antigen and HCG are elevated and help to detect recurrences after operation.^9,10^ However, tumour markers are not seen in the benign type.

Fetal hydrops can occur in the tumour due to high output cardiac failure and anemia which results from arterio-venous shunting and intramural hemorrhage. That is why, fetal hydrops is considered as a prognostic factor for fetuses with SCT and warrants mandatory prenatal careful monitoring.^11^ SCT, being large enough in size, exerts a significant mass effect on adjacent structures during fetal period and results in various co-morbidities such as obstructive hydronephrosis, anterior displacement of the anorectum, bowel distension, hypoplasia of pelvic muscles, mucocolpos or hip dislocation.^7,12^

Although ultrasonography has been the imaging modality of choice during pregnancy as it is a safe method, cost-efficient and widely available, it has few limitations such as limited field of view and difficulties in penetrating bones.^13^ Therefore, in order to overcome the limitations of the ultrasound, CT and MRI are now used for preoperative examination so as to determine the structure, vascularity, exact location, and components of a tumor and its relationship to the surrounding structures.^14,15^ The main treatment for sacrococcygeal teratoma is the complete surgical resection. This can be done by either anterior approach (for tumours extending above the S3 region) or posterior approach (for tumours below S3 region).^16,17^ For large tumours which can not be excised completely can be operated by a combined anteriorposterior approach.^18^ Pre-operative CT and MRI helps in choosing the appropriate path for operative procedure of an SCT.

## Consent:

**JNMA Case Report Consent Form** was signedby the patient and the original article is attached withthe patient's chart.

## Conflict of Interest

**None.**
